# Digital Engagement and Cognitive Function Among Older Adults in China: Cross-Sectional Questionnaire Study and Moderated Mediation Model Analysis

**DOI:** 10.2196/83955

**Published:** 2026-01-20

**Authors:** Yongqi Du, Qing Niu, Gangrui Tan, Jianqian Chao, Shengxuan Jin, Leixia Wang

**Affiliations:** 1 Health Management Research Center School of Public Health Southeast University Nanjing, Jiangsu China

**Keywords:** digital engagement, cognitive function, digital health literacy, social support, living arrangements, moderated mediation model, technological reserve hypothesis

## Abstract

**Background:**

Given the global demographic shifts and rapid digitalization, digital engagement has emerged as a critical determinant of healthy aging. While previous research has linked digital engagement to cognitive outcomes, the underlying mechanisms remain underexplored among Chinese older adults.

**Objective:**

This study aimed to analyze the relationships between digital engagement and cognitive function among older adults in China through a moderated mediation model guided by the technological reserve hypothesis, with digital health literacy (DHL) and social support as mediators and living arrangements as a moderator.

**Methods:**

We conducted a cross-sectional questionnaire survey using stratified multistage sampling from June to November 2024, including 8123 participants aged 55 years and older. Digital engagement, defined as older adults’ use of contemporary digital technologies to support routine daily activities, autonomy, independence, and social inclusion, was assessed using a multidimensional questionnaire. The Chinese eHealth Literacy Scale, the 3-item short version of the Perceived Social Support Scale, and the Mini-Cog test were used to assess DHL, social support, and cognitive function. Guided by a directed acyclic graph based on the technological reserve hypothesis, mediation and moderated mediation analyses were performed using the PROCESS macro in SPSS (IBM Corp) with 5000 bootstrap resamples.

**Results:**

Digital engagement was positively associated with cognitive function among older adults (β=0.241, 95% CI 0.216-0.265). This association was partially mediated by DHL (β=0.059, 95% CI 0.049-0.069) and social support (β=0.012, 95% CI 0.008-0.016), with the combined indirect effects accounting for 29.5% of the total effect (β=0.071, 95% CI 0.061-0.082). Additionally, living arrangements significantly moderated the associations between digital engagement and cognitive function (β=0.109, 95% CI 0.052-0.166), digital engagement and DHL (β=0.063, 95% CI 0.014-0.112), and digital engagement and social support (β=0.151, 95% CI 0.089-0.212). These effects were stronger among older adults living alone.

**Conclusions:**

This study contributes to the understanding of cognitive aging in the digital environment from the perspective of the technological reserve hypothesis and digital engagement. Digital engagement influenced cognitive function via DHL and social support, and these associations of digital engagement with cognitive function, DHL, and social support were stronger among older adults living alone. Digital health interventions and public health policies should target both DHL and social support among older populations and prioritize older adults living alone.

## Introduction

### Background

Cognitive function is a critical determinant of dementia, functional independence, quality of life, and health care burden [1]. With an increasingly aged population, cognitive impairment and dementia have become major health and social issues worldwide [2]. Research has indicated that there are about 15.07 million patients with dementia in the population aged 60 and older in China, while the prevalence of mild cognitive impairment is 15.54%, and the number of patients is 38.77 million [3]. The disease burden of dementia and cognitive impairment is huge. The estimated total annual costs of dementia in China will reach 114.2 billion US dollars in 2030 [4]. Since there is currently no effective pharmaceutical treatment for dementia and mild cognitive impairment, it is important to identify modifiable intermediate risk factors that could prevent cognitive decline [5].

With rapid digitalization and the widespread integration of technology into daily life, digital technologies have emerged as a potential determinant of healthy aging. In China, as of 2025, the number of internet users aged 60 and older has reached 161 million, accounting for nearly 14.4% of all internet users [6]. Many studies have indicated that older people are competent and skilled users of digital technologies [7-9]. Consequently, the concept of digital engagement has been introduced to emphasize the breadth and extent of digital technology use among older people [10]. Every day, digital engagement provides new opportunities for older adults to address age-related cognitive decline. Engagement in cognitively challenging activities, such as learning new digital skills or knowledge, plays a protective role against age-related cognitive decline [11,12]. Meanwhile, access to communication technology and social media facilitates interpersonal interactions and enhances social support [13], which helps maintain cognitive health in older adulthood.

Against this background, exploring the association between digital technology use and cognitive health in later life has become an important research focus. However, the cognitive impact of digital technology use in China has not been sufficiently studied and understood [7,14]. First, research has focused on how access to the internet relates to cognitive function and the associations between use frequency in specific domains and cognitive outcomes [8,15-17]. Many studies in China have investigated the effect of internet or social media use on cognitive function [18,19]. But limited studies give attention to the concept of digital engagement [20] and comprehensively measure the dimensions and frequency of digital technology use. As digital technologies have become increasingly integrated into older adults’ daily lives, it is important to shift research focus from use to meaningful digital engagement to better understand the cognitive effects of digital technology use [7].

Second, although evidence has established the efficacy of digital health interventions for cognitive decline and cognitive impairment, including dementia [21-23], little is known about how they lead to an improvement in symptoms or behavior. The identification of these mediating mechanisms would be useful for tailoring interventions that specifically target these pathways, improving intervention effectiveness. Some studies in China have estimated the mediating roles of physical activity [14] and social support [20,24]. However, few studies have simultaneously examined the roles of multiple mediators. Including multiple mediators can better reflect real-world mechanisms, help understand the relative importance of different intervention pathways, and reduce bias [25].

Third, while digital technologies become increasingly integrated into older adults’ everyday life, growing urbanization and economic reforms in China have transformed intergenerational living arrangements patterns [26]. However, limited studies in China have examined how the association between digital technology use and cognitive function may vary by living arrangements. As the number of older adults living alone in China increases, examining the moderating role of living arrangements in this association is meaningful for developing targeted interventions.

The technological reserve hypothesis provides a theoretical framework for addressing these gaps. This hypothesis, developed by Benge and Scullin, focuses on how digital technology use can counteract cognitive decline and reduce disease burden [27-29]. Technological reserve is defined as “the development of a culture and environment of technology use in older adults that can buffer against the impact of cognitive decline on day-to-day activities” [27]. Further study developed the technological reserve concept and summarized 3 central pathways through which digital technology may prevent cognitive decline [28,30]. First, technology can generate cognitive complexity by engaging older adults in cognitively demanding activities that strengthen cognitive reserve [12,31,32]. By enabling access to diverse information sources (eg, online health information), promoting mentally stimulating activities, and requiring continual learning and adaptation, digital technologies help sustain and challenge cognitive capacities [33]. Second, technology fosters social connection and engagement, which are well-established protective factors against cognitive decline [34]. Through platforms such as social media, messaging apps, and video calls, older adults can maintain social ties, reduce loneliness, and access emotional and instrumental support. Finally, technologies can function as cognitive prosthetics by directly compensating for lapses in memory and executive function, particularly those involved in completing activities of daily living. For example, smartphone apps can deliver reminders for medication adherence [35].

Guided by the technological reserve hypothesis, this study aimed to examine the mediating effect of digital health literacy (DHL) and social support on the relationship between digital engagement and cognitive function, as well as the moderating effect of living arrangements on the relationships among digital engagement, DHL, social support, and cognitive function.

### Theoretical Framework

#### Digital Engagement and Cognitive Function

Within the technological reserve framework, digital technology use as a modifiable lifestyle behavior is a critical factor that can promote better cognitive outcomes than would be expected based on age, brain injury, or disease stage [30]. In this study, we adopted the term “digital engagement” to define digital technology use among older adults. Digital engagement among older adults refers to their use of contemporary digital technologies and devices to carry out routine and enjoyable everyday activities that support autonomy, independence, and social inclusion [8]. This concept emphasizes how older adults integrate information and communication technologies into daily activities and information-seeking behaviors rather than focusing on limitations [36]. Research has investigated the potential association between digital engagement and cognitive function among older adults. Although some studies suggest potential risks such as sleep disruption or social isolation [37-39], the prevailing evidence supports that digital engagement is positively linked to cognitive function [26,40,41]. Empirical findings generally suggest that regular use of digital technologies (such as social media and online social networking) is positively associated with better cognitive outcomes [42-44]. These benefits are often attributed to increased cognitive stimulation, enhanced social connectivity, and greater engagement in mentally active tasks afforded by digital technology [45,46]. Longitudinal studies further suggest that consistent internet use is associated with slower cognitive decline and a lower subsequent risk of dementia compared with nonuse [47,48]. Meta-analyses of randomized controlled trials also support the effectiveness of digital interventions in improving specific cognitive domains [49,50]. Despite growing evidence, the mechanisms through which digital engagement benefits cognition remain insufficiently understood.

#### The Mediating Role of DHL

DHL is the ability to seek, understand, evaluate, and apply health information from digital sources to support health-related decision-making [51]. Within the technological reserve framework, DHL can strengthen cognition by engaging older adults in cognitively demanding processes such as evaluating online resources, learning new digital skills, and applying health information in daily life. These processes involve active learning, adaptive reasoning, and problem-solving, which are consistent with mechanisms that sustain cognitive reserve [30]. Moreover, empirical studies support this pathway. Higher DHL is associated with greater adoption of preventive health behaviors, better management of chronic conditions, improved adherence to treatment, and more informed health decisions [52-55]. Such behaviors not only enhance health outcomes but also contribute to maintaining and preserving cognitive function in later life. Thus, DHL may mediate the association between digital engagement and cognitive function.

#### The Mediating Role of Social Support

Within the technological reserve framework, another plausible pathway operates through social connectivity. Social connectivity refers to the structural and functional aspects of individuals’ social relationships, and in later life, is often reflected through social support received from their networks [56-58]. Socioemotional selectivity theory points out that social participation requires a certain cost investment, and members who engage in social participation are bound to consider cost-benefit issues [59]. For older adults, declining physical and cognitive abilities raise the cost of offline social participation, leading to a gradual reduction in face-to-face interactions [60]. Digital technologies offer alternative and more accessible avenues for maintaining social support [61]. Some empirical studies have shown that digital engagement is positively associated with increased social support in later life [61-63]. Social support, in turn, is a well-established protective factor for cognitive function: Older adults with stronger support networks tend to perform better cognitively and face a lower risk of cognitive decline or dementia [64-66]. Thus, social support may serve as a mediator linking digital engagement to cognitive outcomes.

#### The Moderating Role of Living Arrangements

Economic reforms and urbanization in China since the 1980s have profoundly reshaped family structures, particularly impacting older adults. This shift aligns with modernization theory, predicting smaller families and fewer older adults co-residing with children [67]. Consequently, more older adults live only with a spouse or alone [68]. Given the central role of family in Chinese culture, the study shifted to investigate the moderating role of living arrangements. Within the framework of the technological reserve hypothesis, the cognitive benefits of digital engagement are expected to vary across social contexts that shape baseline access to cognitive and social resources. Living arrangements represent a contextual factor in later life, as co-residence with others may provide routine cognitive stimulation and social interaction, whereas living alone is often associated with reduced offline engagement. Consequently, digital engagement may play a more pronounced compensatory role for older adults living alone by supplementing limited in-person cognitive and social resources. This theoretical perspective provides a rationale for examining living arrangements as a moderator in the association between digital engagement and cognitive function. Additionally, living arrangements may shape both the opportunities and the need for engaging with digital technology [69]. Older adults living alone often rely on digital technologies to maintain social ties, bridge social gaps, and manage independent living [70,71]. In contrast, those in multigenerational households may experience “proxy internet use” (eg, reliance on family members for online tasks), reducing direct engagement and the attendant cognitive stimulation [72]. Digital engagement may therefore be especially protective for those living alone.

### Hypotheses

Guided by the technological reserve hypothesis, this study tested a moderated mediation model to examine whether digital engagement is associated with cognitive function through DHL and social support, and whether these pathways are moderated by living arrangements. Based on the theoretical framework and prior empirical evidence, we propose the following hypotheses:

1. Hypothesis 1: higher digital engagement is correlated with greater cognitive function among older adults.
2. Hypothesis 2: higher digital engagement is associated with higher DHL among older adults.
3. Hypothesis 3: higher DHL is correlated with greater cognitive function among older adults.
4. Hypothesis 4: DHL is a mediator between digital engagement and cognitive function among older adults.
5. Hypothesis 5: higher digital engagement is associated with greater social support among older adults.
6. Hypothesis 6: greater social support is correlated with greater cognitive function among older adults.
7. Hypothesis 7: social support is a mediator between digital engagement and cognitive function among older adults in China.
8. Hypothesis 8: living arrangements moderate the associations of digital engagement with cognitive function, DHL, and social support.

**Figure 1:**
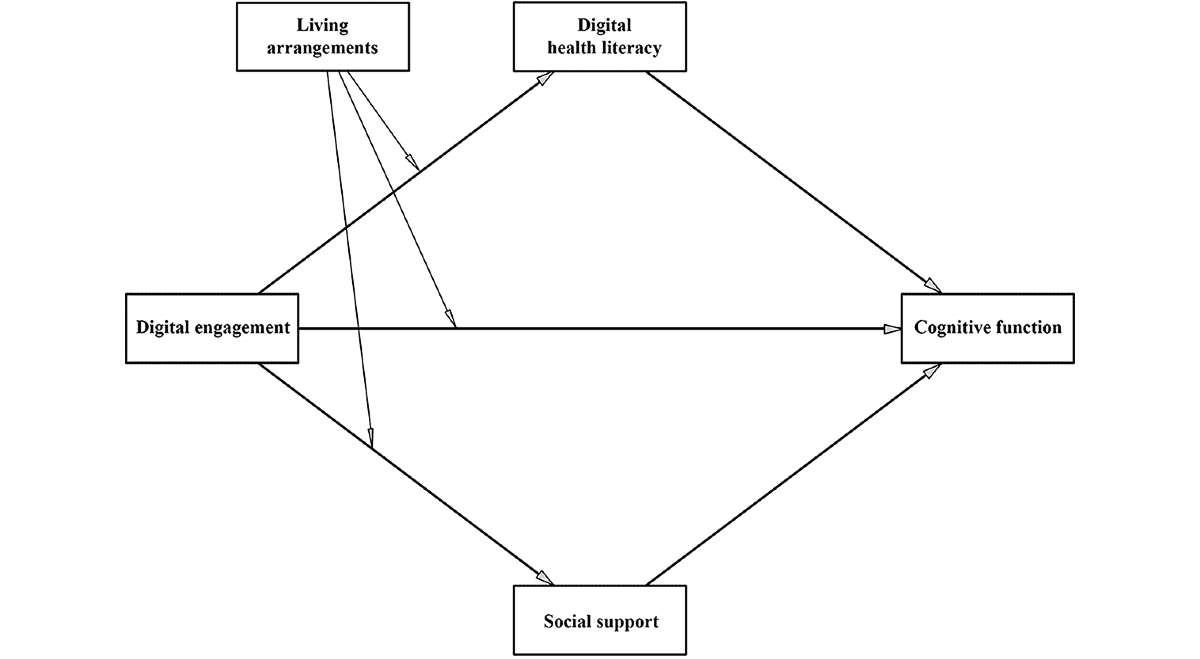
The hypothesized moderated mediation model.

To guide the analyses, we specified a directed acyclic graph (DAG) illustrating the hypothesized relationships among digital engagement, cognitive function, DHL, social support, and living arrangements (Figure 1).

## Methods

### Study Design and Sampling Procedures

This study used data collected through a large-scale, cross-sectional survey conducted concurrently by 5 academic teams affiliated with 4 major universities in China. To ensure methodological uniformity, all participating sites adhered to a unified research protocol during the implementation phase.

A stratified, multistage sampling framework was used to enhance representativeness across regions with different levels of socioeconomic development. China was first stratified into eastern, central, and western regions, which reflect well-documented gradients in economic development, urbanization, and digital infrastructure. One to 2 provinces were randomly selected from each region. The final sample included Hubei (central China), Shandong and Jiangsu (eastern China), and Guangxi (western China), thereby capturing substantial regional heterogeneity in demographic structure and digital development. Within each selected province, 1 to 2 urban or county-level administrative units were further sampled based on local economic conditions, followed by cluster sampling of communities or villages.

Sample size estimation followed the standard formula for proportion-based calculations: 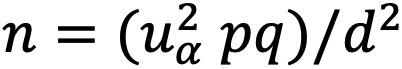, where u_α_ represents the critical value for a 95% CI (u_α_=1.96), p is the estimated proportion of older internet users based on the China Internet Network Information Center’s 51st Statistical Report [73], *q* is the complementary proportion (*q*=1–p), and *d* denotes the allowable error (1.2%). Based on these parameters, the minimum required sample size was calculated as 6616. To account for possible nonresponses and invalid questionnaires, a 20% oversampling rate was applied, resulting in a target sample of approximately 7940 individuals.

### Data Collection and Quality Control

Fieldwork was conducted from June to November 2024 by trained surveyors in collaboration with local village committees or community service offices. Face-to-face interviews were administered at participants’ homes using standardized paper questionnaires. The interviews collected information on sociodemographic characteristics, digital technology use, digital literacy, cognitive function, and quality of life. All surveyors received centralized training to ensure consistent questionnaire administration and interpretation. Upon completion of each interview, field supervisors performed a thorough review of the questionnaires to check for completeness, internal consistency, and data accuracy before submission for entry.

### Participants

**Figure 2:**
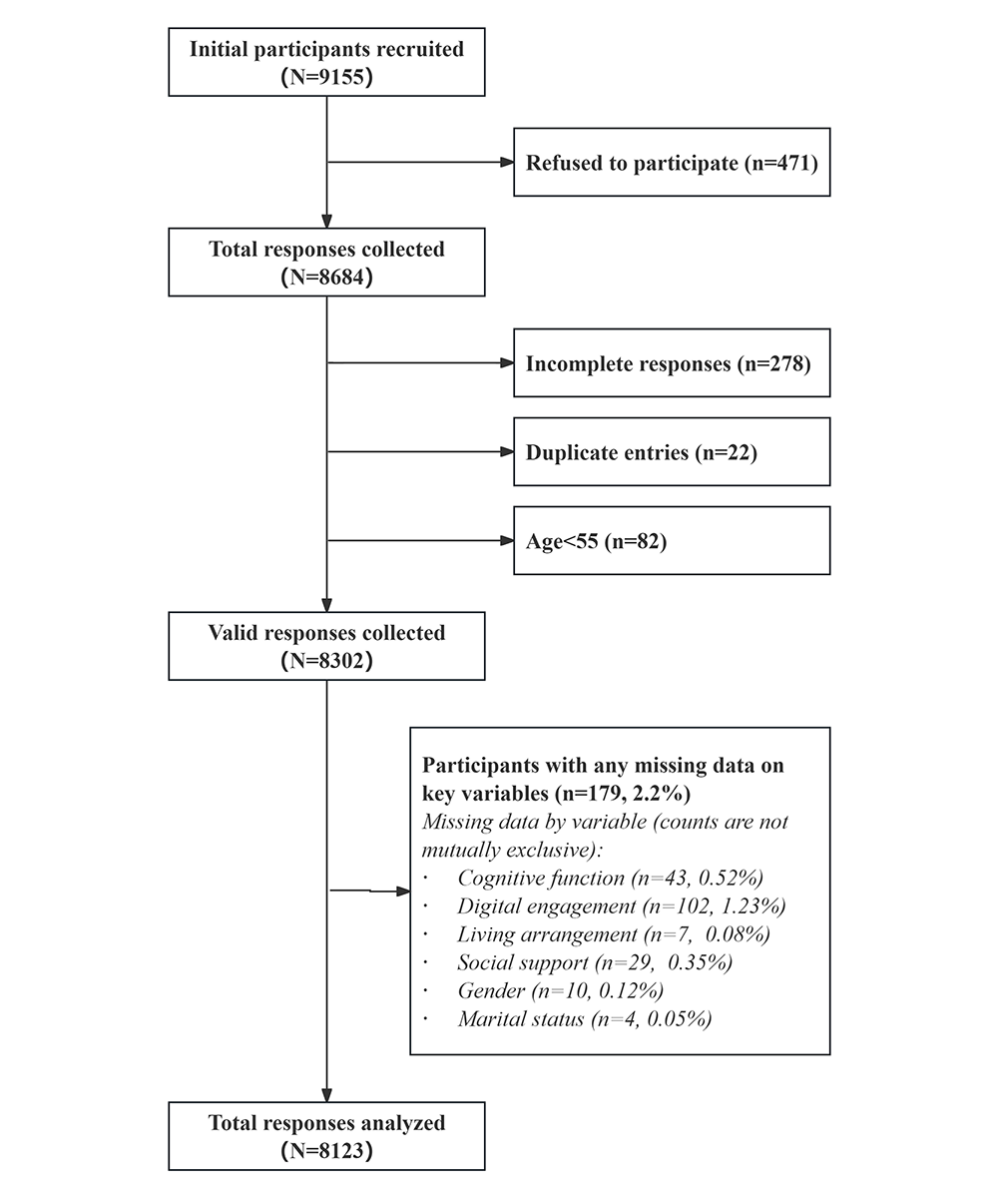
Flow diagram of participant recruitment and data exclusion, resulting in a final analytic sample of N=8123.

Eligible participants were older adults who met the following inclusion criteria: (1) aged 55 years and older, (2) had resided in the sampled community or village for at least 6 months, and (3) were able to communicate effectively with investigators. Exclusion criteria included: (1) individuals temporarily absent from their households during the survey period, (2) those diagnosed with terminal illnesses, and (3) those who declined to participate. After excluding incomplete responses, duplicate entries, and respondents younger than 55 years, 8302 valid questionnaires remained. Among these, 179 participants (2.2%) had missing values on at least 1 analytic variable and were excluded from the main analyses. The final analytic sample consisted of 8123 participants (Figure 2).

### Measurements

#### Cognitive Function

Cognitive function was assessed using the Mini-Cog test. The Mini-Cog test is a rapid, valid, and reliable screening tool for cognitive impairment [74]. The Mini-Cog Test includes a 3-word recall task (scored 0-3) and the clock drawing test (scored 0-2). The total score ranges from 0 to 5. The Mini-Cog test has demonstrated good screening performance in community-dwelling older adults in China [75]. In this study, the Mini-Cog total score was used as a continuous measure of cognitive function, with higher scores indicating better cognitive performance.

#### Digital Engagement

Digital engagement was measured using a self-reported scale developed to capture older adults’ frequency of participation in various digital activities. The scale included eight items of digital behaviors: (1) social communication (eg, using WeChat [Tencent] voice or video calls), (2) experience sharing (eg, posting on WeChat Moments, QQ Zone [Tencent], or Weibo [Sina Corporation]), (3) leisure and entertainment (eg, playing online games, listening to music, or watching videos), (4) online transactions (eg, transferring money, making payments, booking services, or trading stocks), (5) information seeking (eg, searching for travel information or reading news), (6) online learning or training, (7) online civic participation (eg, participating in online polls, petitions, or rights protection), and (8) political engagement (eg, online voting or leaving messages on government websites). Participants rated the frequency of each activity on a 5-point Likert scale ranging from 1 (never) to 5 (always). Higher scores indicated higher digital engagement. The scale demonstrated good internal consistency in this sample (Cronbach α=0.876). Although the scale covers multiple items of digital activities, this study conceptualized digital engagement as an overall behavioral tendency reflecting the breadth and extent of digital technology use in daily life. This approach is consistent with the technological reserve hypothesis and the concept of digital engagement, which emphasize cumulative and sustained engagement. Therefore, a composite digital engagement score was used in the analyses.

#### DHL

DHL was assessed using the eHealth Literacy Scale (eHEALS), a widely validated instrument developed by Norman and Skinner to measure individuals’ self-perceived skills in locating, evaluating, and applying electronic health information to health-related problems [51]. The eHEALS consists of 8 items rated on a 5-point Likert scale (1=strongly disagree to 5=strongly agree), reflecting domains such as awareness of available online health resources, confidence in using the internet for health decision-making, and the ability to discern high-quality digital health content. Given the linguistic and cultural differences between the original instrument and the target population of older adults in mainland China, we used the simplified Chinese version (C-eHEALS) translated and validated by Ma and Wu [76]. The C-eHEALS has been confirmed to have good psychometric properties and can therefore be used to evaluate eHealth literacy in Chinese older populations [77]. In this study, the C-eHEALS demonstrated excellent internal consistency, with a Cronbach α coefficient of 0.986, indicating high reliability for use among Chinese older adults.

#### Social Support

Social support was assessed using the 3-item short version of the Perceived Social Support Scale (PSSS-3), which was developed and validated by Wu et al [78] specifically for use among the Chinese general population. This abbreviated scale was derived from the original 12-item Chinese version of the Multidimensional Scale of Perceived Social Support (MSPSS), originally adapted by Jiang [79] from the version developed by Zimet et al [80]. The PSSS-3 includes 1 item from each of the 3 core dimensions, family support, friend support, and significant others, selected based on the highest factor loadings in a large-scale national sample. The Cronbach α of PSSS-3 in this study was 0.868, demonstrating good internal consistency.

#### Living Arrangements

Living arrangements were measured as a binary variable indicating whether the older adult lived alone, and were assessed using the following question: “What are your current living arrangements?” Those who reported living alone were coded as 1, and those who reported living with others were coded as 0.

#### Control Variables

The prior study indicates that demographic and health factors have close links with cognitive function and suggests that these factors should be included in pertinent research [81]. In this study, gender, age, current place of residence, marital status, education, and number of chronic diseases were controlled as covariates.

### Statistical Analysis

All analyses were performed using SPSS (version 27; IBM Corp). We first examined the extent and pattern of missing data for all analytic variables. The proportion of missing values for each variable ranged from 0.08% to 1.2% and the overall proportion of missing data was 2.2% (Figure 2). Little’s Missing Completely at Random test was conducted using the missing value analysis procedure in SPSS 27. The test indicated that the missing values were independent of the observed or unobserved values (
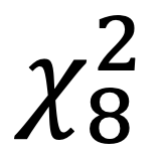
=14.893; *P*=.06). Given the low proportion and completely random patterns of missingness, we performed complete-case analyses based on listwise deletion.

Descriptive statistics summarized sample characteristics, with continuous variables reported as mean (SD) and categorical variables as frequencies and percentages. Pearson correlations examined associations among digital engagement, cognitive function, DHL, social support, and living arrangements. Multicollinearity was assessed by the variance inflation factor (VIF), with VIF>5 indicating collinearity. The relationships among variables were specified according to a DAG based on the technological reserve hypothesis (Figure 1) and analyzed using PROCESS models 4 and 8 with 5000 bootstrapped resamples. Effects were considered significant if the 95% bias–corrected CI did not include 0. All models controlled for age, gender, current place of residence, marital status, education, and number of chronic diseases. Continuous variables were standardized prior to analysis. Statistical tests were 2-tailed with α=.05.

### Ethical Considerations

The study was reviewed and approved by the Medical Ethics Committee of Zhongda Hospital, Southeast University (approval number 2024ZDSYLL294-Y01). Written informed consent was obtained from all participants before they participated in the study, and they were provided with the opportunity to withdraw at any time during and after the survey. To protect privacy and confidentiality, electronic data were de-identified and stored on password-protected devices accessible only to the research team. No images or supplementary materials in this manuscript contain information that could identify individual participants. There was no compensation for the participants in our study survey.

## Results

### Demographic Characteristics of the Participants

The final sample consisted of 8123 older adults, with an average age of 71.03 (SD 8.39) years. Men accounted for 42.46% (3449/8123) of the participants. Among the participants, 3990 (49.12%) lived in urban areas, and 4133 (50.88%) lived in rural areas. Most respondents were married (6338/8123, 78.03%), and 51.73% (4202/8123) of the participants had attained a middle school education or above. Overall, 75.28% (6115/8123) of the participants reported at least 1 chronic condition. Summary statistics for main variables, including digital engagement, DHL, social support, living arrangements, and cognitive function, are presented in Table 1.

**Table 1:** Participant characteristics and descriptive statistics for study variables (N=8123).

Variables			Values
**Sex, n (%)**			
	Male	3449 (42.46)	
	Female	4674 (57.54)	
Age (years), mean (SD)			71.03 (8.39)
**Current place of residence, n (%)**			
	Urban	3990 (49.12)	
	Rural	4133 (50.88)	
**Marital status, n (%)**			
	Married	6338 (78.03)	
	Unmarried	1785 (21.97)	
**Education, n (%)**			
	Primary school or under	3921 (48.27)	
	Middle or high school	3462 (42.62)	
	College or above	740 (9.11)	
**Chronic diseases, n (%)**			
	0	2008 (24.72)	
	1	3477 (42.80)	
	≥2	2638 (32.48)	
Digital engagement, mean (SD)			17.37 (8.03)
DHL^a^, mean (SD)			20.18 (10.59)
Social support, mean (SD)			16.71 (3.22)
**Living arrangements, n (%)**			
	Living alone	1166 (14.35)	
	Living with others	6957 (85.65)	
Cognitive function, mean (SD)			3.57 (1.49)

^a^DHL: digital health literacy.

### Preliminary Correlation Analysis

Table 2 presents the Pearson correlation coefficients among the main variables. Digital engagement was significantly and positively correlated with cognitive function (*r*=0.365, *P*<.001), social support (*r*=0.081, *P*<.001), and DHL (*r*=0.575, *P*<.001), but negatively associated with living arrangements (*r*=–0.101, *P*<.001). Cognitive function was also positively correlated with social support (*r*=0.131, *P*<.001) and DHL (*r*=0.347, *P*<.001), and negatively correlated with living arrangements (*r*=–0.069, *P*<.001). VIFs indicated no multicollinearity among digital engagement (VIF=1.732), DHL (VIF=1.666), social support (VIF=1.027), living arrangements (VIF=1.823), and cognitive function.

**Table 2:** Pearson correlation matrix of digital engagement, digital health literacy, social support, living arrangements, and cognitive function (N=8123).

Variables		Digital engagement	DHL^a^	Social support	Living arrangements	Cognitive function
**Digital engagement**						
	*r*	1	0.575	0.081	–0.101	0.365
	*P* value	—^b^	<.001	<.001	<.001	<.001
**DHL**						
	*r*	0.575	1	0.127	–0.073	0.347
	*P* value	<.001	—	<.001	<.001	<.001
**Social support**						
	*r*	0.081	0.127	1	–0.045	0.131
	*P* value	<.001	<.001	—	<.001	<.001
**Living arrangements**						
	*r*	–0.101	–0.073	–0.045	1	–0.069
	*P* value	<.001	<.001	<.001	—	<.001
**Cognitive function**						
	*r*	0.365	0.347	0.131	–0.069	1
	*P* value	<.001	<.001	<.001	<.001	—

^a^DHL: digital health literacy.

^b^Not applicable.

### Mediation Analysis

Guided by the DAG-specified conditional process model, we first used model 4 of the PROCESS macro for SPSS [82] to test Hypotheses 1 to 7. The total effect of digital engagement on cognitive function was 0.241 (95% CI 0.216-0.265), of which the direct effect accounted for 70.5% (β=0.170, 95% CI 0.143-0.196) and the combined indirect effects by DHL and social support accounted for 29.5% (β=0.071, 95% CI 0.061-0.082). The findings indicated a moderate but statistically meaningful mediation by DHL and social support.

**Figure 3:**
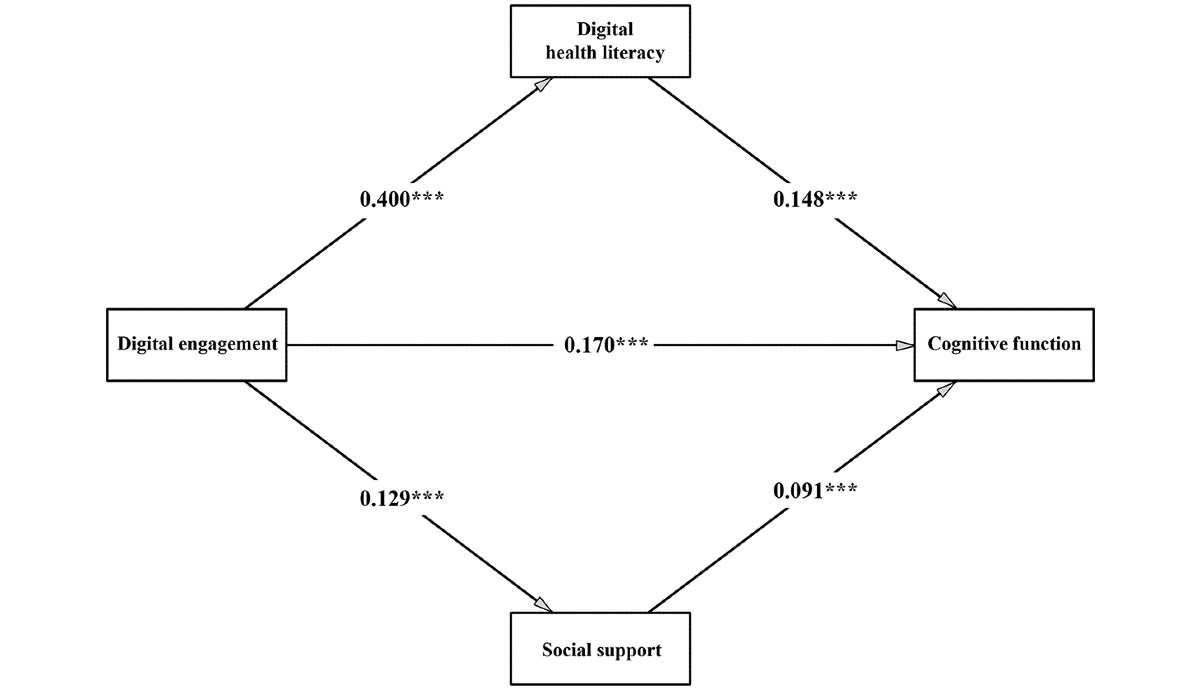
Mediation model of the association between digital engagement and cognitive function through digital health literacy and social support; values are standardized coefficients adjusted for covariates (PROCESS Model 4; N=8123; ***P<.001).

Table 3 and Figure 3 present the results of the mediation analysis. As shown in model 3, the direct effect of digital engagement on cognitive function was significant (Model 3: β=0.170, 95% CI 0.143-0.196; *P*<.001), thus supporting Hypothesis 1. Digital engagement also demonstrated a significant and positive association with DHL (Model 1: β=0.400, 95% CI 0.379-0.420; *P*<.001), supporting Hypothesis 2. Additionally, DHL was significantly and positively related to cognitive function (Model 3: β=0.148, 95% CI 0.123-0.174; *P*<.001), supporting Hypothesis 3. Furthermore, digital engagement was positively and significantly correlated with social support (Model 2: β=0.129, 95% CI 0.103-0.155; *P*<.001), supporting Hypothesis 5. Social support, in turn, showed a significant and positive correlation with cognitive function (Model 3: β=0.091, 95% CI 0.071-0.112; *P*<.001), supporting Hypothesis 6.

**Table 3:** Mediation analysis of the association between digital engagement and cognitive function through digital health literacy and social support, adjusted for covariates (PROCESS Model 4; N=8123).

Model 1^a^ (DHL^b^)					Model 2^c^ (social support)			Model 3^d^ (cognitive function)	
		β^e^ (95% CI)	*P* value	β (95% CI)		*P* value	β (95% CI)		*P* value
**Explanatory variable**									
	Digital engagement	0.400 (0.379 to 0.420)	<.001	0.129 (0.103 to 0.155)		<.001	0.170 (0.143 to 0.196)		<.001
**Mediator variables**									
	DHL	—^f^	—	—		—	0.148 (0.123 to 0.174)		<.001
	Social support	—	—	—		—	0.091 (0.071 to 0.112)		<.001
**Control variables**									
	Gender	–0.035 (–0.070 to –0.001)	.047	0.169 (0.125 to 0.213)		<.001	–0.044 (–0.085 to –0.003)		.03
	Age	–0.041 (–0.060 to –0.021)	<.001	0.065 (0.041 to 0.089)		<.001	–0.095 (–0.117 to –0.072)		<.001
	Current place of residence	0.321 (0.280 to 0.362)	<.001	–0.465 (–0.518 to –0.413)		<.001	0.048 (–0.003 to 0.098)		.06
	Marital status	0.039 (–0.004 to 0.082)	.076	0.159 (0.104 to 0.213)		<.001	0.134 (0.084 to 0.184)		<.001
	Education	0.294 (0.261 to 0.327)	<.001	0.172 (0.131 to 0.213)		<.001	0.179 (0.140 to 0.218)		<.001
	Chronic diseases	–0.124 (–0.147 to –0.101)	<.001	–0.078 (–0.107 to –0.048)		<.001	0.034 (0.007 to 0.062)		.01
Constant		–0.471 (–0.563 to –0.380)	<.001	–0.353 (–0.469 to –0.237)		<.001	–0.383 (–0.491 to –0.275)		<.001

^a^*F*_7, 8115_=811.099; *R*^2^=0.412.

^b^DHL: digital health literacy.

^c^*F*_7, 8115_=65.515; *R*^2^=0.054.

^d^*F*_9, 8113_=214.765; *R*^2^=0.192.

^e^β: standardized regression coefficient.

^f^Not applicable.

These findings suggest that DHL and social support play a partial mediating role in the relationship between digital engagement and cognitive function, with indirect effects of 0.059 (95% CI 0.049-0.069) and 0.012 (95% CI 0.008-0.016), respectively, supporting Hypotheses 4 and 7. Combining 2 mediation effects, the total indirect effect was 0.071 (95% CI 0.061-0.082). The bootstrap test results for indirect effects are reported in Table 4.

**Table 4:** Bootstrap estimates of indirect effects of digital engagement on cognitive function through digital health literacy and social support (PROCESS Model 4; N=8123).

Indirect effects path	Effects	SE	95% CI	Proportion of effects (%)
Total indirect effect	0.071	0.005	0.061-0.082	100
DE^a^→DHL^b^→CF^c^	0.059	0.005	0.049-0.069	83.10
DE→SS^d^→CF	0.012	0.002	0.008-0.016	16.90

^a^DE: digital engagement.

^b^DHL: digital health literacy.

^c^CF: cognitive function.

^d^SS: social support.

### Moderated Mediation Analysis

**Figure 4:**
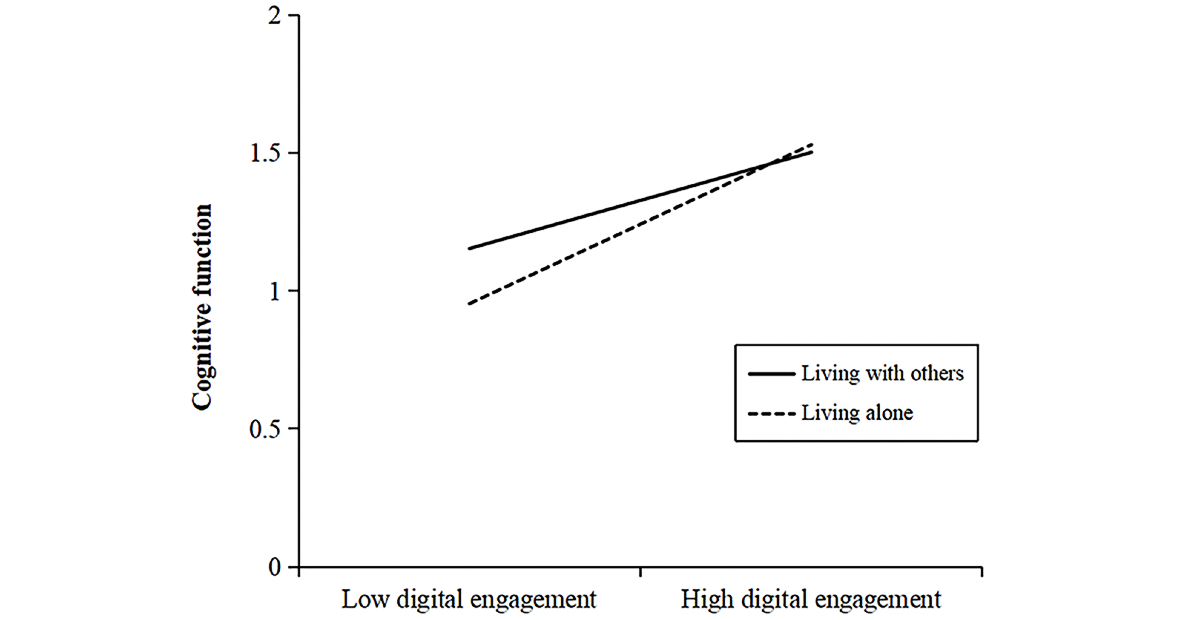
The moderating effect of living arrangements on the association between digital engagement and cognitive function. The difference in simple slopes indicated that the association between digital engagement and cognitive function was significantly stronger for individuals living alone than for those living with others (PROCESS Model 8, N=8123).

To examine the moderated mediation effects involving living arrangements, we used Model 8 of the PROCESS macro for SPSS [82], using 5000 bootstrap resamples and a 95% bias-corrected CI. The results are reported in Table 5. The analysis revealed a significant and positive interaction effect between digital engagement and living arrangements on cognitive function (Model 6: β=0.109, 95% CI 0.052-0.166; *P*<.001), suggesting a moderating role of living arrangements. As illustrated in Figure 4, the beneficial association between digital engagement and cognitive performance was stronger among older adults who lived alone, relative to those who lived with others.

**Table 5:** Moderated mediation analysis testing moderation by living arrangements in the associations between digital engagement and digital health literacy, digital engagement and social support, digital engagement and cognitive function, adjusted for covariates (PROCESS Model 8; N=8123).

Variables		Model 4^a^ (DHL^b^)				Model 5^c^ (social support)			Model 6^d^ (cognitive function)	
		β^e^ (95% CI)	*P* value		β (95% CI)		*P* value	β (95% CI)		*P* value
**Explanatory variable**										
	Digital engagement	0.391 (0.370 to 0.413)		<.001	0.109 (0.082 to 0.137)		<.001	0.156 (0.129 to 0.183)		<.001
**Mediator variables**										
	DHL	—^f^		—	—		—	0.147 (0.121 to 0.172)		<.001
	Social support	—		—	—		—	0.089 (0.069 to 0.110)		<.001
**Moderating variables**										
	Living arrangements	0.054 (–0.011 to 0.119)		.10	0.058 (–0.024 to 0.141)		.17	0.154 (0.078 to 0.230)		<.001
	DE×LA^g^	0.063 (0.014 to 0.112)		.01	0.151 (0.089 to 0.212)		<.001	0.109 (0.052 to 0.166)		<.001
**Control variables**										
	Gender	–0.036 (–0.070 to –0.001)		.044	0.167 (0.123 to 0.211)		<.001	–0.044 (–0.085 to –0.003)		.04
	Age	–0.040 (–0.060 to –0.021)		<.001	0.065 (0.041 to 0.089)		<.001	–0.094 (–0.117 to –0.072)		<.001
	Current place of residence	0.321 (0.280 to 0.362)		<.001	–0.465 (–0.517 to –0.413)		<.001	0.047 (–0.003 to 0.097)		.07
	Marital status	0.063 (0.007 to 0.119)		.03	0.175 (0.103 to 0.247)		<.001	0.210 (0.144 to 0.276)		<.001
	Education	0.292 (0.259 to 0.324)		<.001	0.167 (0.126 to 0.208)		<.001	0.176 (0.137 to 0.215)		<.001
	Chronic diseases	–0.123 (–0.146 to –0.100)		<.001	–0.077 (–0.106 to –0.047)		<.001	0.034 (0.007 to 0.061)		.01
Constant		–0.492 (–0.591 to –0.393)	<.001		–0.360 (0.485 to –0.234)		<.001	–0.456 (–0.572 to –0.339)		<.001

^a^*F*_9, 8113_=632.208; *R*^2^=0.412.

^b^DHL: digital health literacy.

^c^*F*_9, 8113_=53.680; *R*^2^=0.056.

^d^*F*_11, 8111_=178.558; *R*^2^=0.195.

^e^β: standardized regression coefficient.

^f^Not applicable.

^g^DE×LA: the interaction term between digital engagement and living arrangements.

**Figure 5:**
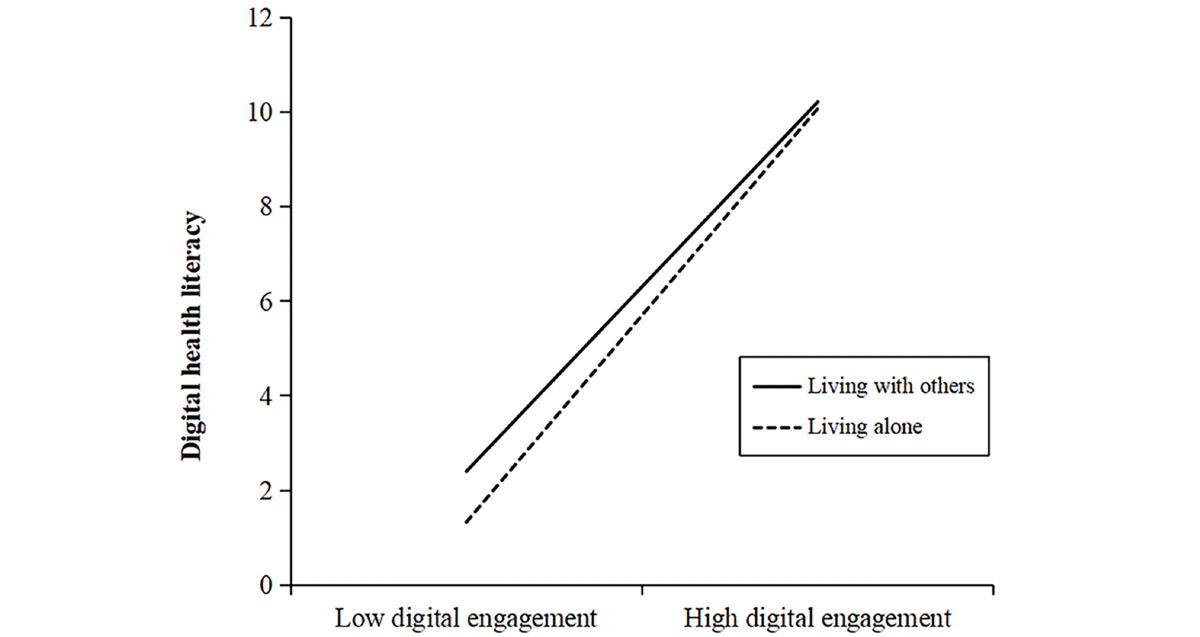
The moderating effect of living arrangements on the association between digital engagement and digital health literacy. The difference in simple slopes indicated that the association between digital engagement and digital health literacy was significantly stronger for individuals living alone than for those living with others (PROCESS Model 8, N=8123).

**Figure 6:**
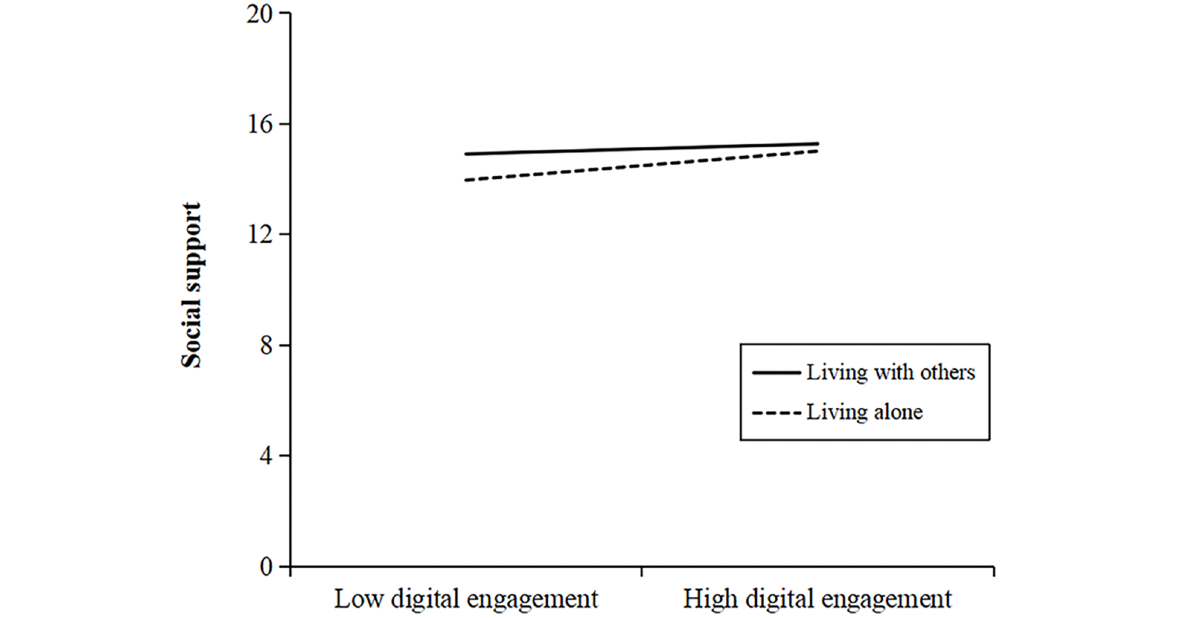
The moderating effect of living arrangements on the association between digital engagement and social support. The difference in simple slopes indicated that the association between digital engagement and social support was significantly stronger for individuals living alone than for those living with others (PROCESS Model 8, N=8123).

In addition to the interaction effect on the direct path, the results also revealed significant moderating effects of living arrangements on the first stages of both mediation pathways. Specifically, the interaction term between digital engagement and living arrangements significantly predicted DHL (Model 4: β=0.063, 95% CI 0.014-0.112; *P*=.01 and social support (Model 5: β=0.151, 95% CI 0.089-0.212; *P*<.001). As illustrated in Figures 5 and 6, the beneficial association of digital engagement with both DHL and social support was stronger among older adults who lived alone, relative to those who lived with others. These findings indicate that living arrangements moderate the associations of digital engagement with cognitive function, DHL, and social support, supporting Hypothesis 8.

Table 6 reports the conditional indirect effects of digital engagement on cognitive function by each path. For the DHL mediator, the indirect effect of digital engagement on cognitive function was 0.057 (95% CI 0.048-0.068) among participants living with others and 0.067 (95% CI 0.054-0.080) among those living alone. The corresponding index of moderated mediation was 0.009 (95% CI 0.003-0.016), indicating a significantly stronger indirect effect by DHL for participants living alone. Similarly, for the social support mediator, the indirect effect was 0.010 (95% CI 0.007-0.013) for those living with others and 0.023 (95% CI 0.016-0.032) for those living alone. The index of moderated mediation for the social support pathway was 0.013 (95% CI 0.007-0.021). In all cases, the 95% CI values excluded zero, indicating that both indirect effects were significantly stronger among older adults living alone.

**Table 6:** Indices of moderated mediation for two conditional indirect effects of digital engagement on cognitive function by living arrangements (PROCESS Model 8; N=8123).

Conditional indirect effects path	Effects	SE	95% CI
DE^a^→DHL^b^→CF^c^ (living with others)	0.057	0.006	0.048-0.068
DE→DHL→CF (living alone)	0.067	0.007	0.054-0.080
Index of the moderated mediation	0.009	0.003	0.003-0.016
DE→SS^d^→CF (living with others)	0.010	0.002	0.007-0.013
DE→SS→CF (living alone)	0.023	0.004	0.016-0.032
Index of the moderated mediation	0.013	0.003	0.007-0.021

^a^DE: digital engagement.

^b^DHL: digital health literacy.

^c^CF: cognitive function.

^d^SS: social support.

## Discussion

### Principal Findings

Guided by the technological reserve hypothesis and using a large, community-based sample of older adults in China, this study investigated the mechanisms underlying the association between digital engagement and cognitive function among older Chinese adults. We found that higher digital engagement was associated with better cognitive performance. DHL and social support partially mediated this association, and the combined indirect effects accounted for 29.5% of this association. Living arrangements moderated both the direct and indirect pathways, with stronger benefits among older adults living alone. These findings extend prior work on technology use and cognition in later life and broaden the application of the technological reserve hypothesis in the Chinese context.

### The Association Between Digital Engagement and Cognitive Function

Digital engagement was significantly and positively associated with cognitive function. This supports the technological reserve hypothesis that digital technology use is a modifiable behavioral factor that can promote better cognitive outcomes [30]. It also aligns with prior work linking internet or computer use to cognition [83-88] and with recent studies in China demonstrating that internet use and digital activities enhance cognitive function [14,47]. By adopting the construct of digital engagement rather than a simple use-versus-nonuse dichotomy, our study advances the field by situating technology use within the everyday life context of older adults, emphasizing how they integrate information and communication technologies into their ongoing activities, social interaction, and information seeking [36]. This finding implies that encouraging sustained and meaningful digital engagement is a promising strategy for public health and aging policies aiming to strengthen cognitive function among older adults.

### The Mediating Role of DHL and Social Support

Consistent with the cognitive-stimulation pathway posited by the technological reserve hypothesis, higher digital engagement was associated with higher DHL, which in turn related to better cognitive performance [28,30]. Notably, the DHL pathway accounted for the majority of the total indirect effect, indicating that health-related digital competencies may be a primary mechanism linking engagement to cognition. Specifically, engaging with digital technology improves DHL because digital skills are among the core skills of DHL [89,90]. In turn, higher DHL denotes a stronger capacity to seek, understand, appraise, and apply health information [91]. These processes involve engaging with cognitively complex information [30], helping older adults build cognitive reserve. This finding advances the eHealth Literacy Model, which posits that DHL is underpinned by cognition [92], and indicates that DHL also serves as a tool that shapes cognition through ongoing, cognitively complex digital activities. Accordingly, interventions should combine user-friendly interfaces with structured, progressive training in cognitively complex digital tasks, ensuring that everyday digital engagement serves as sustained cognitive stimulation. Practical examples include stepwise smartphone or tablet training delivered in community settings (eg, locating health information from reliable sources, evaluating credibility and misinformation, and applying information to everyday self-management tasks).

Social support mediated the association between digital engagement and cognition, consistent with the social-connectivity pathway [28,30]. This finding aligns with previous research showing that digital engagement has the potential to enhance cognitive function among older individuals by addressing feelings of loneliness and improving the social support they receive from relatives and friends [20]. Specifically, digital engagement enables cheap and easy communication between older adults in distant communities, increasing social connections, overcoming social and spatial barriers, and providing a convenient way to stay in touch with families, friends, and the outside world [62]. In turn, better social support is associated with better cognitive outcomes in older adults [65,93-96]. This finding underscores that interventions should help older adults form and maintain digital social ties so that online interactions translate into perceived social support and, ultimately, better cognitive outcomes. For example, programs could incorporate facilitated online peer groups and a “Digital Buddy system” to help older adults translate online interactions into perceived support [97].

### The Moderating Role of Living Arrangements

Our study further revealed that living arrangements played a significant moderating role in the associations of digital engagement with cognitive function, DHL, and social support. Compared with older adults who live with others, those living alone experienced a significantly stronger positive effect of digital technology engagement on cognitive function, consistent with the previous studies [26,98]. This moderating effect was significantly present in both mediating pathways: older adults living alone gained greater benefits in terms of DHL and social support from digital engagement than those living with others. Specifically, older adults living alone, due to a lack of effective offline social interactions, are more reliant on virtual social networks facilitated by digital technologies [60]. This reliance partially compensates for the reduced social support associated with solitary living, thereby mitigating its negative impact on cognitive function [70]. Additionally, older adults who live alone are less likely to engage in proxy internet use [72] and thus rely more on themselves to use digital devices (eg, searching for health information online). Furthermore, since older adults living alone are less often burdened with caregiving responsibilities for grandchildren, they have more freedom and time to engage with digital technologies [26]. Our findings suggest that digital engagement serves as a more efficacious strategy for mitigating cognitive decline among older adults living alone compared to those living with others.

Our finding is broadly consistent with other international evidence. A cohort study in America reported that transitioning into Internet use was associated with better cognitive function and slower cognitive decline, and that these benefits were more pronounced among older adults living alone than among those living with others [84]. Additionally, a 2-country longitudinal study in Sweden and the Netherlands observed less decline in global cognition among baseline internet users after adjustment for living situation [86]. Beyond cognitive outcomes, findings based on the Survey of Health, Ageing and Retirement in Europe further indicate that internet use can attenuate the association between living alone and loneliness across different European welfare regimes, implying that digital engagement may buffer psychosocial vulnerabilities of solitary living [99]. Taken together, although the prevalence and social meaning of living alone differ across cultures, converging evidence supports that digital engagement may serve as a compensatory resource for older adults with constrained offline or household-based support.

This moderation finding has practical implications for intervention design. Digital inclusion initiatives to help older people adapt to digital technologies should prioritize this vulnerable group. An integrated community-based approach may be especially useful: individual digital coaching (eg, guided practice in health information seeking) coupled with structured social support (eg, online group chats). Including a “living alone” priority within such initiatives may help maximize equity and potential cognitive benefits, while also addressing social isolation risks that have been recognized as a public health and policy concern.

### Limitations and Future Research

Despite these contributions, several limitations should be acknowledged. First, the cross-sectional design precludes causal inferences. Although our models adjusted for a set of covariates, endogeneity, reverse causation, and unmeasured confounding cannot be fully ruled out. Thus, our results only show associations, not causality. Future studies should use longitudinal designs with extended follow-up periods to elucidate temporal dynamics and disentangle potential reverse causation. Second, reliance on self-reported measures introduces the risk of recall bias, especially among participants with cognitive impairments, despite our use of validated instruments to attenuate this issue. Third, digital engagement was operationalized as a composite measure. Thus, this study could not disentangle potentially differential effects of specific types of digital activities on cognitive function. Finally, due to data constraints, living arrangements were operationalized as solitary versus nonsolitary living, precluding differentiation among various household compositions (eg, living with a spouse, children, or extended family). Given the important role of family structures in the well-being of older adults in China, future research should refine classifications of living arrangements to better explore their moderating effects.

### Conclusions

This study contributes to the understanding of cognitive aging in the digital environment from the perspective of the technological reserve hypothesis and digital engagement. First, it offers an innovative framework based on the technological reserve hypothesis for understanding the moderating and mediating mechanisms of DHL, social support, and living arrangements. Second, it advances previous assessment methods of digital technology application by using a comprehensive measure. Our results increase understanding of the mechanisms underlying the cognitive effects of digital technology use and provide insights for designing digital health interventions and public health policies.

## Abbreviations

C-eHEALS: Chinese version of the eHealth Literacy Scale
DAG: directed acyclic graph
DHL: digital health literacy
eHEALS: eHealth Literacy Scale
MSPSS: Multidimensional Scale of Perceived Social Support
PSSS-3: 3-item short version of the Perceived Social Support Scale
VIF: variance inflation factor

## Data Availability

The datasets generated or analyzed during this study are not publicly available due to restrictions under existing Data Use Agreements, but are available from the corresponding author on reasonable request.
